# Could Dietary Goals and Climate Change Mitigation Be Achieved Through Optimized Diet? The Experience of Modeling the National Food Consumption Data in Italy

**DOI:** 10.3389/fnut.2020.00048

**Published:** 2020-05-04

**Authors:** Marika Ferrari, Luca Benvenuti, Laura Rossi, Alberto De Santis, Stefania Sette, Deborah Martone, Raffaela Piccinelli, Cinzia Le Donne, Catherine Leclercq, Aida Turrini

**Affiliations:** ^1^Research Centre for Food and Nutrition, Council for Agricultural Research and Economics, Rome, Italy; ^2^Department of Computer, Control, and Management Engineering Antonio Ruberti, Sapienza University of Rome, Rome, Italy

**Keywords:** dietary intakes, linear programming, greenhouse gas emissions, food consumption, diet optimization, healthy and sustainable diet, nutritional recommendations, sustainable development goals

## Abstract

**Objective:** The aim of this study is to define a healthy and sustainable diet model with low GHGE, fulfilling dietary requirements, and considering current Italian food consumption patterns.

**Design:** A duly designed database was developed, linking food nutritional composition and GHGE based on 921 food items consumed in Italy according to the last national food consumption survey (INRAN-SCAI 2005–2006). Linear programming was used to develop new diet plans separately for males and females, aged 18–60 years (*n* = 2,098 subjects), in order to minimize GHGE. The program is based on dietary goals and acceptability constraints as well as on 13 nutrient requirement constraints aiming to reach a healthy and acceptable diet for the Italian population.

**Results:** Diet optimization resulted in a nutritionally adequate pattern minimizing GHGE values (4.0 vs. 1.9 kg CO_2_e/day for males and 3.2 vs. 1.6 kg CO_2_e/day for females). In both sexes, the nutrient intake of the optimized diet was at the established lower bound for cholesterol and calcium and at the established upper bound for free sugar and fiber. In males, intake of zinc was at the established lower bound whereas iron was at the established upper bound. Consumption of red meat and fruit and vegetables was at the established lower and upper bound, respectively, in both males and females. Despite the decrease in meat consumption, especially red meat, in the optimized diet with respect to the observed diet, levels of iron intake in females increased by 10% (10.3 vs. 11.3 mg/day) but remained below the adequate intake established in Italian national DRIs.

**Conclusions:** An attainable healthy dietary pattern was developed that would lead to the reduction of GHGE by 48% for males and by 50% for females with respect to current food consumption in the Italian adult population. Health-promoting dietary patterns can substantially contribute to achieve related Sustainable Development Goals.

## Introduction

An adequate and balanced diet contributes to achieve a good state of health and to prevent chronic diseases (1, 2). Most high-income countries, Italy among them, develop their country-specific nutrient-based recommendations, referred to as Dietary Reference Intake (DRIs), for assessment and planning of adequate dietary intake (3–5). Healthy food consumption patterns meeting these requirements can be promoted through the development of Food-based Dietary Guidelines, which help to maintain high consumption of local and culture-specific foods (6, 7).

Industrialized countries are facing a wide range of diet-related non-communicable diseases, obesity among population, and micronutrient deficiency. Moreover, due to unbalanced dietary profiles, the DRIs are far from being met for some key nutrients (8). The observation of current food consumption patterns shows that a healthier diet could be obtained in the Italian population through an increase in consumption of vegetable source foods such pulses, fruits, and vegetables and a decrease of consumption of red and processed meat (9, 10).

In addition to nutrition and health considerations, there is an increasing concern regarding the environmental impact of food production as *diets are inextricably link human health and environmental sustainability* (11). Therefore, the achievement of the 12th Sustainable Development Goal (SDG), i.e., “responsible consumption and production” necessarily implies changes in the food consumption pattern. Moreover, the diet is referred to, directly or indirectly, in many “Sustainable Development Goals” (6), including Defeating hunger (2nd), Health and well-being (3rd), Quality education (4th), Consumption and responsible production (12th), and Fight against climate change (13th) (12). Notwithstanding complexity of sustainable diets (13), more and more countries are developing Food-Based Dietary Guidelines that allow promoting diets that are both healthy and sustainable (14, 15) and recently, a conceptual framework to evaluate sustainability in dietary guidelines has been published (16). It is indeed estimated that, globally, food system accounts for 19 to 29% of all greenhouse gas emissions (GHGE), considered to be the principal driver of climate change (17). In Italy, emissions from the agricultural sector (with the exclusion of energy used for agricultural purpose) account for 6.9% of total national GHGE, which is the second source of emissions after the energy sector (81.8%) (18).

The goal set by the European Commission is to reduce domestic emissions by 40% to 2030, compared to baseline data of 1990, in order to mitigate climate change (19). The reduction of GHGE could be achieved through changes in food consumption habits, since production of the same quantities of different foods is responsible for different levels of greenhouse gas, with livestock having the highest emission level (20). Dietary recommendations dealing with environmental sustainability typically focus on limiting consumption of animal-based products (21–23). Alongside, public health authorities recommend plant-based diets for health benefits, such as a lower risk of non-communicable diseases (24), and a limited amount of animal products, especially red and processed meat, in order to decrease health risk, such as colon cancer (25). A review of epidemiological studies on the environmental impact of diets suggested that dietary changes aimed to reduce diet-related GHGE may also promote health (14, 26). A research project on food and nutrition systems has evidenced that consuming a healthy and balanced diet increases nutrition-related parameters, allows for stabilizing climate, increases biodiversity conservation, reduces a little bit of productivity, but increases value added (27).

A possible approach to avail of commonalities between healthy diets and diets with low environmental impact is to design optimized diets in the context of multiple constraints through linear programming. Diet optimization by linear programming is a mathematical approach that optimizes (minimizes or maximizes) a linear function of decision variables while complying with multiple constraints. Use of linear programming to solve the so-called “diet problem” in order to find a solution joining a healthy diet and a low-cost diet started in 1945 when Jerry Cornfield started to study this topic for the Army in the Second World War (28). Linear programming has been used for decades for informing nutrition; in fact, diet was one of the first problems on which optimization method was tested by Dantzig (29). Recently several food-based dietary guidelines have been developed based on optimized healthy diets (30).

Previous studies have also suggested that diet optimization methods can be useful to develop a nutritionally adequate diet with low GHGE, while maintaining the social and cultural preferences of the population, by taking the mean population dietary intake as the baseline (31, 32). The research focuses on estimating a dietary pattern with low environmental impact while maintaining the social and cultural preferences of the population (33). Macdiarmid et al. (34) published a diet that meets dietary requirements with low GHGE without ruling out meat or dairy products from the diet. An interesting study (35) showed that a theoretical vegetarian diet does not reduce GHGE more than an optimized omnivore diet.

Another issue of diet optimization considering low GHGE regards the need for standardized environmental data for linear programming. In most high-income countries, including Italy, country-specific food composition databases are available to determine the nutrient composition of food consumption patterns. No similar country-specific standardized environmental food databases of GHGE are available. Studies providing GHGE data for some foods (36, 37) do exist, but these data are not comparable with one another, since the methodologies are not standardized. Hence, there is a need to integrate and to standardize an environmental food database (38) also in Italy.

In addition, only a few studies on optimized diet minimizing GHGE are available for Italy, since they resulted from a European perspective (37) or they concern some specific target groups of a small and no representative sample (39).

The principal aim of this study was to develop an optimized diet for the adult Italian population, thus defining a nutritionally optimal food consumption pattern able to meet the national DRIs and with the minimum GHGE. Then, a linear programming approach based on the last dietary data from INRAN-SCAI 2005–2006 food consumption database has been applied. To this purpose, a GHGE database for a wide range of foods items was created from literature considering Italian specific data. The secondary aim of the present study is to investigate the impact of an optimized diet, likely to be low in meat and dairy products, in terms of the coverage of requirements for selected nutrients for which these products are important contributors in the current Italian diet.

## Materials and Methods

### Dietary Data

The survey methodology to collect the most recently published nationwide food consumption data has been described by Leclercq et al. (9). The national cross-sectional survey (INRAN-SCAI 2005–2006) had been carried out by the National Research Institute for Food and Nutrition (INRAN) on a random sample of the Italian population. The studied sample consisted of 3,323 individuals belonging to 1,329 households (1,501 males and 1,822 females, aged 0.1–97.7 years). The adult subsample analyzed in the present study was composed of 18–60-year-old individuals (*n* = 2,098 subjects), a homogeneous group of adults for the nutritional recommended range values (4).

A 3-day semi-structured diary was used for participants to record all their food and drink consumption in addition to their intake of nutritional supplements and of medicines containing nutrients.

Individual intakes of foods were calculated with the use of the software INRAN-DIARIO version 3.1 (9). The INRAN-SCAI 2005–2006 food database included 1,119 food items and 123 dietary supplement foods. The food composition database in terms of nutrients has been described by Sette et al. (40) and used for the present study. The selected subset of foods for the present analysis amounts to 921 food items, with the exclusion of infant formula (198 food items) and dietary supplement foods because infant population was not considered and GHGE values for supplements are currently inconsistent.

The classification of food items was performed at the ingredient level as reported by Leclercq et al. (9) and was aggregated into 15 food categories and 55 subcategories. Appendix 1 provides a description of the consumed food items.

Weighted mean nutrient content (energy, protein, total fat, SFA, PUFA, carbohydrates, cholesterol, free and intrinsic sugars, fiber, calcium, iron, zinc, and vitamin B_12_) have been calculated by each food category and subcategory for males and females separately. These values were standardized to 100 g in order to be used in the linear model.

### GHGE Data

GHGE values, expressed as kilograms CO_2_ equivalents (kg CO_2_e), were identified from national and international published Life Cycle Assessment (LCA) studies or green literature (conference papers, project report, technical sheet) with similar system boundaries that included food processing, distribution, and retailing. More than 50 scientific articles were considered to extract values of GHGE for individual foods. A summary of GHGE literature references on food items used in the present study is reported in Appendix 2. More than 50% of these articles refer to studies developed in Italy. Eighteen Italian technical sheets of environmental product declaration were used in the calculation of GHGE of some food products (Appendix 2). The assessment of combined impact of different greenhouse gases, such as methane (CH_4_) or nitrous oxide (N_2_O), was achieved using Global Warming Potential (GWP) assuming a 100-year perspective, where the emission expressed as CO_2_e (Carbon dioxide equivalent) allows one to describe different greenhouse gases in a common unit; e.g., 1 kg of CH_4_ is equal to 25 kg of CO_2_e and 1 kg of N_2_O is equal to 298 kg of CO_2_e (41).

A total number of 102 GHGE values of food products named as “indicator products” was identified from the literature (Appendix 2). Appendix 1 specifically shows the indicator products considered for each category and subcategory. Each indicator product for which data points identified were matched to similar food items in the subcategory is indicated in the footnotes. In some cases, it was considered enough that one indicator product represented several items consumed in the same food subcategory. For instance, orange juice GHGE value was applied for all types of fruit and vegetable juices (e.g., nectar, carrot juice, orange juice), or cola GHGE value from a specific brand was applied for all types of carbonate beverages (e.g., cola, soda, ginger ale, orange, tonic water).

### Mean Values and Uncertainty Ranges of GHGE Data

For each indicator product, the GHGE value was obtained as the mean of the data points; then, the GHGE value for each subcategory was calculated by averaging the GHGE values of the component indicator products.

In addition to calculating mean values of GHGE, uncertainty ranges were produced for this study but not used for the optimization to address their great variability both within and between similar food items. For example, the GHGE variation per kilogram of food item was 0.6–2.4 kgCO_2_e for milk and yogurt, 1.4–12.3 kgCO_2_e for meat (12.3–18.6 kgCO_2_e for beef), and 0.8–18.9 for cheese (2.6–9.1 kgCO_2_e for hard cheese). The same principles proposed by Hartikaiinen and Pulkkinen (42) were used and adapted for this study in order to develop a database:

- *Quartiles*: whenever four or more data sources from literature were available, we used the lower quartile as the minimum value of uncertainty range and the upper quartile as the maximum value.
- *minimum–maximum*: whenever only 2–3 data sources were available, we used minimum and maximum values.
- *unique value 50-50*: if there was only one GHGE estimate, we used −50%–+50% uncertainty range, unless the estimate was a national evaluation, but it was considered reasonably reliable according to international estimates of similar products; in this case, the range was −25%–+25%.

### Linear Programming Model

A linear programming model has been developed to minimize GHGE while satisfying a set of nutritional, acceptability, and healthy constraints. GHGE is a linear function of the 55 food subcategory amounts (*x*_1_, *x*_2_, …, *x*_55_), i.e.,

$$\begin{array}{l} GHGE=\overset{55}{\underset{k=1}{\sum }}c_{k}x_{k} \end{array}$$

where *c*_*k*_ is the GHGE per *g* of food category *k*. Diets are constructed to minimize the objective *GHGE*, while satisfying the following constraints.

### Nutritional Constraints

Nutrient and energy constraints were derived from national dietary reference values (4) in terms of established lower and upper bound values for total energy, proteins, total fat, saturated fatty acids (SFA), polyunsaturated fatty acids (PUFA), carbohydrates, free and intrinsic sugar, cholesterol, fiber, vitamin B_12_, iron, calcium, and zinc. The choice of iron, calcium, zinc, and vitamin B_12_ as micronutrients was related to the fact that their coverage in Italian diet is critical in some classes of ages and physiological conditions, such as iron in women in childbearing age. In addition to that, processed meat and milk products are key sources of these micronutrients. It is therefore expected that a lower content of these animal foods in an optimized low carbon footprint diet might jeopardize the coverage of nutrient requirements for these micronutrients.

Free and intrinsic sugar recommendation was defined according to WHO (43) that considers free sugar as all sugars added to foods or drinks by the manufacturer, cook, or consumer, as well as sugars naturally present in honey, syrups, fruit juices, and fruit juice concentrates. Free sugars have different physiological significance from the so-called intrinsic sugars, which are those incorporated within the structure of intact fruit and vegetables, and sugars from milk (lactose and galactose). We therefore calculated free sugar from foods such as biscuits, cakes, snacks, milk-based desserts, yogurt with sugars, candies, chocolates, alcoholic and soft drinks, and fruit juices and preserved fruits with sugar excluding fresh fruits and vegetable and milk and natural yogurts.

Different values were considered for males and females, respectively, as shown in Tables 1, 2.

**Table 1:** Nutritional constraints for daily intake compared with the mean observed diet (INRAN-SCAI 2005–2006) and the optimized diet from linear programming model for the adult male population, 18–60 years^a^.

	**Established lower and upper bound**	**Observed diet**	**Optimized diet**
**Nutritional**			
GHGE^b^ (kgCO_2_e)		4.0	1.9
Energy (kcal/day)	2,400–2,460	2,406	2,400
Protein (g/day)	60–92	93.2	77.9
Total fat (% Energy)	24.5–30.8	36.0	30.5
SFA^c^ (% Energy)	7.0–10.1	11.2	8.8
PUFA^d^ (% Energy)	4.8–10.1	4.6	5.5
Cholesterol (mg/day)	250–300	334.0	250.0
Carbohydrates (% Energy)	46.8–65.7	47.5	60.2
Free + intrinsic sugar (% Energy)	10.4–16.3	13.2	14.0
Free sugar (% Energy)	4.2–5.5	7.9	5.5
Fiber (g/day)	24–26	19.5	26.0
Calcium (mg/day)	900–1,100	801.0	900.0
Iron (mg/day)	9–11	12.6	11.0
Zinc (mg/day)	11–13	12.7	11.0
Vitamin B_12_ (μg/day)	2–3	6.7	2.6
Alcohol (g/day)	0	13.4	0.0
Fruit and vegetables (g/day)	400–500	423.0	500.0
Red meat (g/day)	10–30	73.0	10.0
Processed meat^e^ (g/day)	0	36.0	0.0
**Cultural acceptability**			
Total weight of food (g/day)	1,825–3,193 (80–140) % of the total weight of the mean observed diet	2,281	2,941
^f^Food categories and subcategories	5th ≤ and ≤ 90th percentile calculated on the mean observed diet^a^^,^^c^	(see Table 3)	

a Non-consumers included;

b Greenhouse Gas Emission;

c Saturated Fatty Acids;

d polyunsatured Fatty Acids;

e The term “processed meat” refers to meat (usually red meat) preserved by smoking, curing, or salting, or by addition of preservatives. Meat preserved only by refrigeration, however they are cooked, are usually not classified as “processed meat.”

f *Except for pulses and fish where the quantities were established as ≥20 g/day*.

**Table 2:** Nutritional constraints for daily intake requirements compared with the mean observed diet (INRAN-SCAI 2005–2006) and the optimized diet from linear programming model for the adult female population, 18–60 years^a^.

	**Established lower and upper bound**	**Observed diet**	**Optimized diet**
**Nutritional**			
GHGE^b^ (kgCO_2_e)		3.2	1.6
Energy (kcal/day)	1,900–1,982	1,947	1,900
Protein (g/day)	52–73	76.3	67.3
Total fat (% Energy)	24.1–31.3	36.8	28.8
SFA^c^ (% Energy)	6.4–10.4	11.4	9.2
PUFA^d^ (% Energy)	4.5–10.4	4.7	4.7
Cholesterol (mg/day)	250–300	266.0	250.0
Carbohydrates (% Energy)	46.0–66.7	48.7	60.7
Free + intrinsic sugar (% Energy)	10.3–16.6	17.8	15.4
Free sugar (% Energy)	4.0–5.5	8.3	5.5
Fiber (g/day)	24–26	17.5	26.0
Calcium (mg/day)	900–1,100	729.0	900.0
Iron (mg/day)	17–19^e^	10.3	11.3
Zinc (mg/day)	8–10	10.6	10.0
Vitamin B_12_ (μg/day)	2–3	5.6	2.6
Alcohol (g/day)		4.8	
Fruit and vegetables (g/day)	400–500	420.0	500.0
Red meat (g/day)	10–30	54.0	10.0
Processed meat^f^ (g/day)		24.0	
**Culturally acceptability**			
Total weight of food (g/day)	1,670–2,923 (80–140)% of the total weight of the mean observed diet	2,088	2,900
^g^Food categories and subcategories	5th ≤ and ≤ 90th percentile calculated on the mean observed diet^a^^,^^c^	(see Table 3)	

a Non-consumers included;

b Greenhouse Gas Emission;

c Saturated Fatty Acids;

d Polyunsaturated Fatty Acids;

e This range was not used as explained in the Results section. After the first running of the model, it was observed that the maximum value of iron compatible with all other constraints is 11.8 g/day;

f The term “processed meat” refers to meat (usually red meat) preserved by smoking, curing, or salting, or by addition of preservatives. Meat preserved only by refrigeration, however they are cooked, are usually not classified as “processed meat.”

g *Except for pulses and fish for which quantities were fixed as ≥20 g/day*.

### Acceptability Constraints

The mean total amount of food and beverage intake was constrained to range between 80 and 140% of the observed mean intake (2,281 g/day for men and 2,088 g/day for female) in order to comply with acceptability of the diet. The upper limit was increased with respect to the value of 120% used by Perignon et al. (26) to compensate for total removal of some food categories such as processed meat and alcoholic beverages in the diet. Moreover, food category and subcategory quantities were constrained to be within the 5th and the 90th percentile of the observed food consumption. Percentiles were calculated by gender.

### Healthy Constraints

In order to ensure that optimized quantities of some food groups that are usually either promoted or restricted for a sustainable diet are in accordance with international recommendation for healthy diet, established lower and upper bounds were set as constraints for fruits and vegetable and alcohol beverages categories and for red meat and processed meat subcategories.

The term “red meat” refers to beef, pork, horse, lamb, and goat from domesticated animals. The amount was calculated based on the International Agency for Research on Cancer (IARC) (24) recommendation for cancer prevention corresponding to <400 g per week of raw weight of red meat. For these reasons, the constraint used in this paper was restrictive (<30 g/day) in consideration of the benefits for general public health prevention in relation to other non-communicable diseases. Fruit and vegetables do not include pulses and fruit juices. The recommended amount for fruit and vegetable (at least 400 g/day) is derived from WHO/FAO (44). A zero upper limit was imposed to the model for alcoholic beverages (45) and processed meat according to international recommendations (24).

Moreover, a minimum intake of pulses and fish at least of 20 g/day was added in consideration of the high importance of consumption of these food groups in the context of a balanced diet (4, 6).

All the considered constraints were expressed as linear functions of food subcategory amounts as follows:

$$\begin{array}{l} lb_{j}\leq \overset{55}{\underset{k=1}{\sum }}a_{jk}x_{k}\leq ub_{j} \end{array}$$

The values *lb*_*j*_ and *ub*_*j*_ define the established lower and upper bounds for the *j*th constraint and *a*_*jk*_ are either the contribution of the food subcategories per unit weight (DRIs) or are all equal to 1 (acceptability of total weight) or assume value 1 or 0 when referring to constraints on single categories or subcategories (5th and 90th percentiles or recommended values).

For the subcategory “processed meat” and the category “alcoholic beverages,” the recommendation present in the Italian national dietary guidelines (6) is to avoid consumption. Therefore, the corresponding subcategories were constrained to have a zero consumption. Despite these values being very far from the mean, they are still realistic since the 5th percentile of the observed consumption is equal to zero.

### Statistical Analysis

Descriptive statistics (mean, 5th and 90th percentile) were calculated for male and female separately using the Statistical Analysis System computer software package (SAS package version 9.01; SAS Institute Inc., Cary, NC).

Optimization was performed using the solver for linear programming of the Optimization Toolbox™ of Matlab® (https://www.mathworks.com/products/optimization.html).

## Results

At the first stage of application, linear programming provided a solution for male but could not provide a solution for female population data. This means that there exists no diet satisfying all the constraints no matter the GHGE level. The comparison of the sets of constraints for male and female population suggests that infeasibility may depend on the much higher level of iron requested for females. Removing the iron constraint makes the model feasible. Consequently, in order to identify the highest possible iron intake compatible with all other constraints, iron intake was maximized while satisfying the remaining set of nutritional, acceptability, and healthy constraints. A maximum values of mean iron intake of 11.8 mg/day was obtained in females. This means that a diet satisfying all the constraints (apart that for iron) cannot have a mean intake of iron >11.8 mg/day. Hence, the constraint on iron intake related to 17–19 mg/day (4) was not considered (Table 2).

The model resulted into an optimized diet based on 13 nutrient constraints and well-defined acceptability constraints. The mean reduction of GHGE was 4.0 vs. 1.9 kg CO_2_e/day for male and 3.2 vs. 1.6 kg CO_2_e/day for female. Tables 1, 2 show results by males and females comparing the observed diet with the optimized diet.

In terms of nutrients, the observed diet showed that fiber intake for male and female resulted in covering <80% of the recommendation in the observed diet; SFA and vitamin B_12_ in both males and females and cholesterol intakes only in male exceeded the recommended upper limits. Protein requirement (PRI value) referred to the Italian national DRI^4^ and was covered by 83% of the total sample of adults (data not shown in the table).

The optimized diets are slightly different for males and females. In both sexes, the daily intake of the optimized diet was at the established lower bound for cholesterol and calcium, and at the established higher bound for free sugar and fiber. In males, the optimized diet was also at the established lower bound for zinc intake and at the established higher bound for iron intake. In addition to that, the optimized diet was at the established higher bound for fruit and vegetable consumption (500 g/day) (Tables 1, 2) whereas red meat was at the established lower bound (10 g/day) in both males and females.

The daily intake for food categories and subcategories of the optimized diet is reported in Table 3 for males and females. Nine subcategories for males and females belonging to the “potatoes and crisps,” “canned fruit,” “meat substitute,” “offal, blood and their product,” “milk-based dessert and substitute,” “cacao and cacao-based powder,” “artificial sweeteners,” “meal substitute,” and “other fats” groups have not reached admissible solutions because of the low consumption level by the adult sample. The optimized diet is rich in carbohydrates, fiber, and total fat since the intake of pasta and butter for males and potatoes, nuts, milk, and yogurt for both males and females is very similar or equal to the 90th percentile of observed current consumption.

**Table 3:** Food categories and subcategories of daily portion of the observed and optimized diets by the adult 18–60-year-old male and female population^a^.

	**Categories**		**Subcategories**	**Observed diet (g/day)**						**Optimized diet (g/day)**	
				**Males**			**Females**			**Males**	**Females**
				**5th**	**90th**	**Mean**	**5th**	**90th**	**Mean**		
1	Cereals, cereal products, and substitutes			130.1	438.7	298.8	98.9	345.5	233.2	388.1	298.3
		1	Bread and flour	29.6	262.8	150.2	11.9	188.0	103.9	186.7	174.9
		2	Pizza	0.0	33.3	9.1	0.0	33.3	7.8	0.0^c^	0.0^c^
		3	Breakfast cereals	0.0	0.0	1.0	0.0	7.0	1.9	0.0^d^	0.0^c^
		4	Pasta with eggs, filled, etc.	0.0	13.5	4.5	0.0	10.8	3.3	13.5^b^	10.8^b^
		5	Pasta, pasta substitute and flour	16.7	124.7	78.4	12.4	106.1	63.5	124.7^b^	49.9
		6	Rice	0.0	43.5	16.5	0.0	39.5	15.2	21.0	0.0^c^
		7	Biscuits	0.0	42.2	13.8	0.0	35.3	12.6	42.2^b^	35.3^b^
		8	Cakes and sweet snacks	0.0	54.9	19.7	0.0	50.0	16.2	0.0^c^	0.0^c^
		9	Savory fine bakery products	0.0	20.0	5.7	0.0	27.4	9.0	0.0^c^	27.4^b^
2	Pulses			0.0	38.6	11.3	0.0	36.9	11.2	20.0	36.9^b^
		10	Pulses, fresh or processed	0.0	38.6	11.3	0.0	36.9	11.2	20.0	36.9^b^
3	Vegetables			75.0	384.2	231.5	71.8	339.3	211.3	306.8	329.3
		11	Leafy, fruiting and other vegetables, fresh	35.6	301.1	167.9	37.0	271.9	157.6	248.0	271.9^b^
		12	Roots and onions, fresh	1.1	53.6	19.7	0.3	52.8	19.1	53.6^b^	52.8^b^
		13	Vegetables, processed	0.0	91.1	41.8	0.0	73.8	32.6	0.0^c^	0.0^c^
		14	Spices and herbs	0.0	5.2	2.1	0.0	4.7	1.9	5.2^b^	4.7^b^
4	Potatoes and tapioca			0.0	135.9	55.0	0.0	119.3	46.6	133.0	116.8
		15	Potatoes and potato-based dishes and tapioca, excl. crisps	0.0	133.0	54.2	0.0	117.3	46.1	133.0^b^	116.8
		16	Potatoes crisps	0.0	0.0	0.8	0.0	0.0	0.5	0.0^d^	0.0^d^
5	Fruit			0.0	390.4	194.6	0.0	399.7	211.6	193.2	170.7
		17	Citrus and stone fruits, fresh	0.0	368.3	174.0	0.0	380.0	191.5	183.9	162.3
		18	Exotic fruits	0.0	66.7	17.4	0.0	58.3	17.2	0.0^c^	0.0^c^
		19	Nuts, seeds, dried fruit, olives and their products	0.0	9.3	3.1	0.0	8.4	2.6	9.3^b^	8.3
		20	Fruit canned	0.0	0.0	0.1	0.0	0.0	0.2	0.0^d^	0.0^d^
6	Meat, meat products, and substitutes			40.8	220.7	132.6	24.4	166.7	97.9	40.8^c^	24.4^c^
		21	Beef and veal, not preserved, excl. offal	0.0	102.3	49.5	0.0	85.5	38.0	0.0^c^	0.0^c^
		22	Pork, not preserved, excl. offal	0.0	57.6	16.4	0.0	46.7	11.5	10.0	10.0
		23	Poultry and game, not preserved, excl. offal	0.0	65.5	22.5	0.0	51.8	18.8	30.8	14.4
		24	Processed meat	0.0	80.0	36.1	0.0	54.0	23.7	0.0^e^	0.0^e^
		25	Other meats, not preserved, excl. offal	0.0	33.3	6.8	0.0	0.0	4.9	0.0^c^	0.0^d^
		26	Meat substitute	0.0	0.0	0.0	0.0	0.0	0.1	0.0^d^	0.0^d^
		27	Offals, blood, and their product	0.0	0.0	1.2	0.0	0.0	0.8	0.0^d^	0.0^d^
7	Fish and seafood			0.0	122.8	48.8	0.0	113.5	45.0	20.0	20.0
		28	Crustaceans, shellfish, mussels	0.0	69.0	16.6	0.0	55.2	13.4	0.0^c^	0.0^c^
		29	Fish, fresh	0.0	68.2	25.0	0.0	67.9	25.5	20.0	20.0
		30	Fish, preserved	0.0	23.5	7.3	0.0	22.5	6.2	0.0^c^	0.0^c^
8	Milk, milk products, and their substitutes			21.7	345.8	177.9	24.3	345.6	194.3	311.9	345.6^b^
		31	Milk, milk-based beverages	0.0	240.8	93.8	0.0	250.0	111.3	240.8^b^	250.0^b^
		32	Yogurt and fermented milk	0.0	50.0	16.1	0.0	93.7	27.0	50.0^b^	92.2
		33	Milk based dessert and substitute	0.0	0.0	1.3	0.0	0.0	1.1	0.0^d^	0.0^d^
		34	Cheese and substitutes	3.8	125.8	66.7	3.1	102.6	54.9	21.0	3.4
9	Oils and fats			21.5	68.1	46.0	18.2	56.9	39.0	52.1	30.4
		35	Olive oil	15.3	55.1	36.6	12.6	47.3	31.3	24.0	12.6^c^
		36	Other vegetable oil	0.0	8.8	3.2	0.0	6.7	2.7	8.8^b^	5.3
		37	Butter, creams	0.0	14.7	4.9	0.0	12.5	4.0	14.7^b^	12.5^b^
		38	Other fats	0.0	4.7	1.3	0.0	3.3	0.9	4.7^b^	0.0^c^
10	Sweet products and substitutes			2.7	79.2	36.9	0.0	67.7	31.3	19.6	19.5
		39	Ice cream, popsicle and substitutes	0.0	33.3	11.1	0.0	33.3	8.9	2.2	8.6
		40	Chocolate and substitutes	0.0	7.5	2.4	0.0	6.7	2.1	0.0^c^	6.7^b^
		41	Sugar, fructose, honey, and other nutritious sweeteners	0.0	38.3	19.6	0.0	33.8	16.2	17.3	0.0^c^
		42	Candies, jam, and other sweet products	0.0	10.0	3.3	0.0	13.3	3.6	0.0^3^	4.2
		43	Cacao and cacao-based powder	0.0	0.0	0.5	0.0	0.0	0.5	0.0^d^	0.0^d^
		44	Artificial sweeteners	0.0	0.0	0.0	0.0	0.0	0.1	0.0^d^	0.0^d^
11	Meal substitute			0.0	0.0	0.1	0.0	0.0	0.0	0.0^d^	0.0^d^
		45	Meal substitute	0.0	0.0	0.1	0.0	0.0	0.0	0.0^d^	0.0^d^
12	Eggs			0.0	59.0	24.3	0.0	48.2	18.8	20.4	29.8
		46	Eggs	0.0	59.0	24.3	0.0	48.2	18.8	20.4	29.8
13	Non-alcoholic beverages			251.2	1435.2	858.9	331.2	1478.4	888.0	1435.2^b^	1478.4^b^
		47	Tap water (as such, in beverages or recipes)	0.0	586.7	175.0	0.0	640.0	196.0	586.7^b^	640.0^b^
		48	Mineral water	0.0	986.7	478.3	0.0	1040.0	498.3	841.9	836.6
		49	Herbal tea, tea, coffee, and substitutes (decaffeinated)	6.7	266.7	135.4	1.8	271.7	137.9	6.7^c^	1.8^c^
		50	Fruit and vegetable juices (without artificial sweetener)	0.0	125.0	32.0	0.0	133.3	31.2	0.0^c^	0.0^c^
		51	Other soft drinks	0.0	113.3	38.1	0.0	106.7	24.6	0.0^c^	0.0^c^
14	Miscellaneous			0.0	8.0	3.3	0.0	8.0	3.3	0.0^c^	0.0^c^
		52	Miscellaneous	0.0	8.0	3.3	0.0	8.0	3.3	0.0^c^	0.0^c^
15	Alcoholic beverages			0.0	386.7	161.1	0.0	173.4	56.7	0.0^e^	0.0^e^
		53	Regular wine and substitute	0.0	280.1	101.4	0.0	120.1	35.6	0.0^e^	0.0^e^
		54	Sweet wine, spumante, wine-based appetizers % liquor	0.0	13.3	4.5	0.0	0.0	2.0	0.0^e^	0.0^e^
		55	Beer, cider, and substitute	0.0	166.7	55.1	0.0	66.7	19.1	0.0^e^	0.0^e^

a Non-consumers included;

b Portion of food at 90th percentile;

c Portion of food at 5th percentile equal to 0 except for herbal tea, coffee, and substitute;

d Set to zero since both 5th and 90th percentile are equal to zero:

e *Set to zero in order to avoid the consumption*.

The model resulted in consumption of “spices and herbs” subcategory and “tap water” at the 90th percentile and reduced portion of meat was established according to the healthy constraints in the optimized diet.

Some food categories were not considered to assess the acceptability of the optimized diet because their 90th percentile in the observed diet was zero. This is the case for “breakfast cereals” in males and for “potatoes crisp”, “canned fruit”, “meat substitute and offal, blood and their products”, “milk-based dessert”, “cacao and cacao-based powder”, “artificial sweeteners” and “meal substitute” for both males and females.

The optimized and observed consumption of total fruit were similar in males' diet, with a higher proportion of “citrus fruit and other fruits” but neither “exotic fruit” nor “fruit, canned” in the optimized diet. The total consumption of vegetables was higher in optimized diet vs. the observed diet, particularly due to a higher consumption of leafy and fruiting vegetables and of roots and onion. Consumption of processed vegetables was zero (for both males and females) in the optimized diet.

The optimized consumption for some food subcategories showed minor changes from the observed consumption (within plus or minus 20% of the observed diet). It is the case, for example, in both males and females for “fresh fish.” Conversely, “herbal tea, tea, coffee and substitute (decaffeinate)”, volumes decrease to the lower level at the 5th percentile. The level of meat consumption fits the lower allowable level (5th percentile of the population eats 40.8 g/day or more for males and 24.4 g/day or more for females) with an increase of the quantity of poultry for males (22.5 g/day for observed diet vs. 30.8 g/day for optimized diet). The optimized diet includes 10.0 g/day of pork meat in both males and females, corresponding to the established lower bound of the healthy constraint set for red meat.

**Figure 1:**
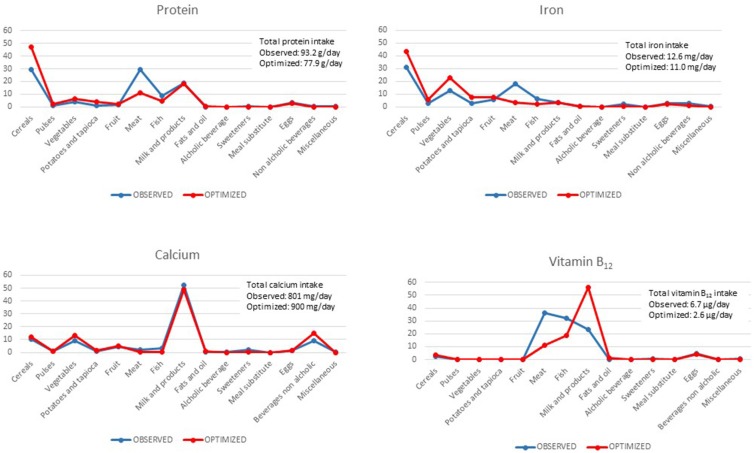
Percentage contribution of food categories to total intake of selected nutrients (protein, iron, calcium, and vitamin B_12_) in observed and optimized diet in the male adult population.

**Figure 2:**
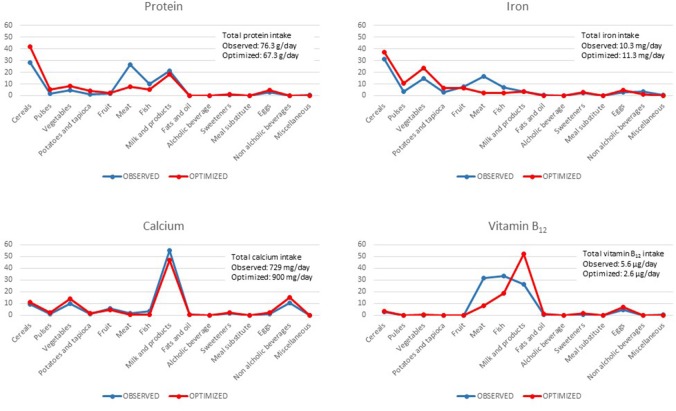
Percentage contribution of food categories to total intake of selected nutrients (protein, iron, calcium, and vitamin B_12_) in observed and optimized diet in the female adult population.

The optimized diet resulted in the minimum recommended intake for animal food products as fish and meat, for both males and females. This results in trade-offs to comply with nutrient constraints on quantities of specific nutrients. In particular, the optimized diet limiting meat category emphasizes the importance of other food categories. In both males (Figure 1) and females (Figure 2), this concerns (i) “cereals” as sources of proteins, (ii) “beverages” as sources of calcium, and (iii) “eggs” and “milk and yogurt” categories as sources of vitamin B_12_.

## Discussion

The present study shows how nutrient-based recommendations proposed by national DRI (4) can be transformed into a practical dietary advice among the Italian adult population, using a linear programming optimization model and the national dietary intake data. To our knowledge, this is the first exercise of applying a mathematical model for diet optimization, using national food consumption data linked to a country-specific GHGE database. In order to provide a healthy and acceptable diet, the optimization, besides minimizing the GHGEs, considers various kinds of constraints: nutrient coverage, acceptability, and health promotion. Nutritional requirements are based on country-specific recommendations (4) from which were derived adequate range values for energy and nutrients intake. Acceptability constraints on food quantities were established to ensure that the optimized diet remained within the range of diet consumed by the Italian population, and were introduced by limiting the food subcategories consumption within the 5th and 90th percentiles of the observed population data. Health constraints led to a complete removal processed meat and alcohol from the diet since they are classified as carcinogen in humans according to IARC (24, 46). The issue of iron intake in optimized diet for females requires a specific consideration. At the first stage of application of linear programming optimization to female population data, no solution was found. This means that there exists no diet satisfying all the constraints no matter the GHGE level. Iron requirement in female compared with usual intake is a critical issue. Italian female diet is low in terms of iron intake (9), and in some groups of population, there is evidence of moderate clinical deficiencies (47). The reason for not obtaining a diet model compatible with iron recommendation in females is related to the partial incompatibility between this specific nutritional constraint and the more general health constraint. If the red meat constraint had been removed, a higher iron intake would have been reached in females. The present study shows that an acceptable diet, nutritionally adequate, and health promoting, is also compatible with a positive mitigation of climate impact. The climate impact of the optimized diet resulted, indeed, to be lower than that of mean Italian dietary intake, that is, 1.9 vs. 4.0 kg CO_2_e/day for males, and 1.6 vs. 3.2 kg CO_2_e/day for females. This means a reduction of emissions of 43% for males and for 50% females with acceptable changes in food consumption pattern and attaining nutrient recommendations.

Macdiarmid et al. (34) performed a similar study on UK female population data obtaining a 36% of GHGEs reduction by imposing nutritional and acceptability constraints. Moreover, the optimized diet required a shift from meat and high-fat and sweet food toward fruit and vegetables as well as starchy food. Meat consumption was reduced by 60% with respect to the national dietary levels while the consumption of dairy products remained unchanged.

Optimizing a diet according to the healthy constraints determines a reduction of meat consumption that in some cases (e.g., processed meat) implies a drastic dietary change, a very long-term process for the population. However, our data demonstrated that it is possible to optimize a diet including limited quantity of red meat (10.0 g/day for both males and females with a reduction of 85 and 80%, respectively, with respect to currently observed levels of consumption). The increase of poultry consumption for males (37%), necessary for the GHGE optimization, is also relevant from a healthy point of view (4, 6) as dietary guidelines suggest a shift from red meat to white meat, including poultry.

Despite the decrease in meat consumption, especially red meat, in the optimized diet, levels of iron intake in females increase by 10% (10.3 vs. 11.3 mg/day). This is possible because of the increment in the intake of pulses, vegetables, and cereals that are important food sources of iron, as observed by Sette et al. (48). The percentage of heme iron—absorbable form without recommended ranges of values—decreased in the diet optimized (3%) compared to that observed (10%) (data not shown in the table); therefore, adult females should adopt special attention in the choice of vegetables and foods rich in dietary enhancer (for example, vitamin C) of non-heme iron absorption (49). As previously reported, using an iron recommendation (4) (18.0 mg/day) for females did not make it feasible to find a solution for an optimized diet. This was a direct effect of the assumption that optimized diet should be acceptable, meaning not too different from the assessed habits. Italian adult females have a very low intake of iron (Table 2), well below the recommendation, so that calculation leading to an important increase in iron intake is not compatible with the model.

Cheese consumption decreases 69% (males) and 90% (females) in optimized vs. observed diet: a potential important modification that is however in line with recommendations considering that Italian people should reduce their consumption from five portions per week (9) to three portions per week (4). In the optimized diet, spices and herbs resulted in the 90th percentile in both males and females. This result represents a further convergence between requirements to achieve climate change mitigation and health outcome. In fact, several food-based dietary guidelines, including the Italians ones, pointed out the importance of replacing salt with spices and herbs as a strategy to reduce the incidence of high blood pressure and related diseases. In parallel, it is recommended to vary choices within spices and herbs in order to limit exposure to toxic components, e.g., methyl-eugenol in basil (50), naturally present in herbal products. This study provides a further contribution to previously similar researches, as it fosters a healthy diet with low GHGE without the total elimination of meat and dairy foods from the diet. On the other hand, Italian habits are preserved as imposed by the acceptability constraints. In fact, the optimized diet proposes many basic foods that are usually included in the meal of the Italian population (pasta, potatoes, vegetables, nuts, milk, and yogurt). In addition, this study provides an evolution of the national food consumption database including also GHGE values for individual food processing, distribution, and retailing. Emissions after the retail phase, such as transports to the household, storing, and cooking, were not included, as well as waste management. On the other hand, a study performed in the UK estimating consumer-specific GHGEs indicated that transport to home, storage at home, preparation, and disposal might represent 16% of total food-related emissions and 2.7% of all GHGE (51). GHGE data in the present paper were developed as much as possible with country-specific estimations; in fact, more than 50% of GHGE data come from scientific or gray literature of heterogeneous studies, conducted under different LCA modeling hypotheses. Anyway, Bertoluci (38) showed that a database of GHGE values estimated with a hybrid method, combining input/output and LCA approaches with a dataset retrieved from literature, improves the quality of data for building a standardized representative national GHGE database for food products. Another possible limitation of this study may be that data collection occurred more than 10 years ago (2005–2006) and therefore they could not represent correctly current Italian dietary habits. Hence, the real effort to adopt the proposed healthy and environment friendly diet could be greater or lower than the one estimated in this study, depending on the increase/decrease of food consumption in certain food groups. This study, however, has established a valid methodology that can be used when updated GHGE values and new national food consumption data will be available.

Moreover, we must consider that comparing global trend at the main food group level has shown a generalized decrease of per-capita intakes (g/day), including meat intake, while an increase of fish and seafood and composite (semi- or fully ready-to-eat products) food intakes had been observed across the 1980–1984 (52), 1994–1996 (53), and 2005–2006 (9) surveys. This appears to confirm the feasibility of the optimization here carried out so the results are suitable to analyze future survey results to understand whether observed diet will converge to optimized values or not.

The present results are in line with indicators of compliance to policy goals for a sustainable and nutritionally valid food system (54) also highlighted in Europe by the Food Agenda 2030 (55). This is important because, to some extent, the paradigm of changing food system for environmental protection is reversed considering that the first action to achieve an impact on sustainability is to follow nutrition recommendations (56). Analysis to foreseen food systems scenarios in 2030 and 2050 also evidenced consumers to play a central role in shaping a future sustainable agri-food system (57, 58). These first results encourage research in extending optimization incorporating further aspects in a multidisciplinary concept of diet sustainability (59, 60) and possibly food safety (61) in a worldwide context (62) linking nutrition and food system (63) in benchmarking Italian dietary patterns (27, 33).

## Conclusion

Diet optimization, using linear programming model, can translate nutrient-based recommendations into acceptable dietary patterns for Italian adult population determining moreover a positive mitigation of climate impact. In particular, a GHGE reduction of around 43% for males and 50% for females was obtained.

The most important results of this study are the alignment of healthy dietary patterns with climate change indicators and an acceptable selection of foods within the eating habits having less environmental impact while complying with nutritional needs. In fact, evidence from this paper suggests that dietary pattern with a reduced environmental impact in terms of GHGE is compatible with a healthy and acceptable diet for the Italian population; dietary patterns that adhered to dietary guidelines (as a whole, not only in part) were more sustainable than the population's current mean amount of dietary pattern intake. These results can be used as a pillar around which optimization is extended to incorporate further aspects in a multidisciplinary concept of diet sustainability in a country-specific context to consider social organizations, economic structures, and cultures.

Overall, these results support the hypothesis that pursuing diet-related goals can substantially contribute to achieving SDGs.

## Data Availability Statement

All datasets generated for this study are included in the article/Supplementary Material.

## Author Contributions

MF, LB, LR, AD, SS, and DM have substantially contributed to conception and design. MF, LB, AD, and SS have given substantial contributions to acquisition of data and/or analysis. MF, LB, LR, AD, SS, and DM have given substantial contributions to interpretation of data. MF was responsible for article writing. MF, LB, LR, AD, SS, DM, RP, CLD, CL, and AT have contributed and everyone has given final approval of the version to be submitted and any previous revised version.

## Conflict of Interest

The authors declare that the research was conducted in the absence of any commercial or financial relationships that could be construed as a potential conflict of interest. The reviewer FV declared a past co-authorship with several of the authors MF and SS to the handling Editor.

## Abbreviations

AR: Average requirement
CH_4_: Methane
CO_2_: Carbon dioxide
CO_2_e: Carbon dioxide equivalent
DRIs: Dietary Reference Intake values
GHGE: Greenhouse gas emissions
GWP: Global warming potential
IARC: International Agency for Research on Cancer
INRAN: National Research Institute for Food and Nutrition
SCAI: Food Consumption Study in Italy
LCA: Life cycle assessment
N_2_O: Nitrous oxide
PRI: Population reference intake
PUFA: Polyunsaturated fatty acids
SDGs: Sustainable Development Goals
SFA: Saturated fatty acids
WHO: World Health Organization.

## References

[1] World Health Organization Nutrient Requirements and Dietary Guidelines, Healthy Diet. (No. 394) [Fact sheet]. Geneva: WHO; Department of Nutrition for Health and Development (2018).

[2] Shrivastava SR Shrivastava PS Ramasamy J. World health organization advocates for a healthy diet for all: global perspective. J Res Med Sci. (2016) 21:44. 10.4103/1735-1995.183994 27904590 PMC5122184

[3] Institute of Medicine Food and Nutrition Board. Dietary Reference Intakes; Applications in Dietary Assessment. Washington, DC: The National Academy Press (2000).

[4] Società Italiana di Nutrizione Umana Livelli di Assunzione di Riferimento di Nutrienti ed Energia per la Popolazione Italiana (LARN) IV Revisione. In: SICS Editore S.r.l., Coordinamento editoriale Milan: SINU INRAN (2014).

[5] Consensus Study Report of The National Academies of Sciences Engineering Medicine - Committee on the Development of Guiding Principles for the Inclusion of Chronic Disease Endpoints in Future Dietary Reference Intakes Guiding Principles for Developing Dietary Reference Intakes Based on Chronic Disease. Washington, DC: The National Academy Press (2017).

[6] Istituto Nazionale di Ricerca per gli Alimenti e la Nutrizione Linee Guida per una Sana Alimentazione Italiana. Revision. Rome (2003).

[7] European Food Safety Authority Scientific Opinion on establishing Food-Based Dietary. EFSA Panel on Dietetic Products, Nutrition, and Allergies (NDA). EFSA J. (2010) 8:1460 10.2903/j.efsa.2010.1460

[8] Mozaffarian D. Dietary and policy priorities for cardiovascular disease, diabetes, and obesity: a comprehensive review. Circulation. (2016) 133:187–225. 10.1161/CIRCULATIONAHA.115.018585 26746178 PMC4814348

[9] Leclercq C Arcella D Piccinelli R Sette S Le Donne C Turrini A. The Italian national food consumption survey INRAN-SCAI 2005-06. Main results in terms of food consumption. Public Health Nutr. (2009) 12:2504–32. 10.1017/S1368980009005035 19278564

[10] Martone D Roccaldo R Censi L Toti E Catasta G D'Addesa D. Food consumption and nutrient intake in Italian schoolchildren: results of the ZOOM8 study. Int J Food Sci Nutr. (2013) 64:700–5. 10.3109/09637486.2013.775226 23480239

[11] Willett W Rockström J Loken B Springmann M Lang T Vermeulen S. Food in the anthropocene: the EAT–lancet commission on healthy diets from sustainable food systems. Lancet. (2019) 393:447–92. 10.1016/S0140-6736(18)31788-4 30660336

[12] United Nations Sustainable Development Goals. Available online at: https://sustainabledevelopment.un.org/sdgs (accessed November 18, 2019).

[13] Tuomisto HL. The complexity of sustainable diets. Nat Ecol Evolut. (2019) 3:720–1. 10.1038/s41559-019-0875-5 30988495

[14] Gonzales Fisher C Garnett T Plates, Pyramids and Planets. Developments in National Healthy and Sustainable Dietary Guidelines: A State of Play Assessment. Oxford: Food and Agriculture Organization of the United Nations (FAO); Food Climate Research Network (FCRN); FAO and the University of Oxford (2016).

[15] Lang T Mason P Sustainable diet policy development: implications of multi-criteria and other approaches, 2008-2017. Proc Nutr Soc. (2017) 4:1–16. 10.1017/S0029665117004074 29198210

[16] Ahmed S Downs S Fanzo J Advancing an integrative framework to evaluate sustainability in national dietary guidelines. Front Sustain Food Syst. (2019) 3:76 10.3389/fsufs.2019.00076

[17] Vermeulen SJ Campbell BM Ingram JIS Climate change and food systems. Annu Rev Environ Resour. (2012) 37:195–222. 10.1146/annurev-environ-020411-130608

[18] Istituto Superiore per la Protezione e la Ricerca Ambientale Italian Greenhouse Gas Inventory 1990-2015, National Inventory Report. Rome: ISPRA, Rapporti 261 (2017).

[19] European Commission 2030 Climate & Energy Framework. Available online at: https://ec.europa.eu/clima/policies/strategies/2030_en (accessed April 17, 2020).

[20] Stehfest E Bouwman L van Vuuren D den Elzen MGJ Eickhout B Kabat P Climate benefits of changing diet. Clim Chang. (2009) 95:83–102. 10.1007/s10584-008-9534-6

[21] Reynolds CJ Buckley JD Weinstein P Bolan J. Are the dietary guidelines for meat, fat, fruit and vegetable consumption appropriate for environmental sustainability? A review of the literature. Nutrients. (2014) 6:2251–65. 10.3390/nu6062251 24926526 PMC4073148

[22] Scarborough P Allender S Clarke D Wickramasinghe K Rayner M. Modelling the health impact of environmentally sustainable dietary scenarios in the UK. Eur J Clin Nutr. (2012) 66:710–5. 10.1038/ejcn.2012.34 22491494 PMC3389618

[23] Tilman D Clark M. Global diets link environmental sustainability and human health. Nature. (2014) 515:518–22. 10.1038/nature13959 25383533

[24] International Agency for Research on Cancer Red meat and processed meat. In: IARC Monographs on the Evaluation of Carcinogenic Risks to Humans. Vol. 114 Lyon: IARC Working Group on the Evaluation of Carcinogenic Risks to Humans (2018).

[25] Givens DI. Review: dairy foods, red meat and processed meat in the diet: implications for health at key life stages. Animal. (2018) 12:1709–21. 10.1017/S1751731118000642 29606182

[26] Perignon M Vieux F Soler LG Gabriel Masset G Darmon N. Improving diet sustainability through evolution of food choices: review of epidemiological studies on the environmental impact of diets. Nutr Rev. (2017) 75:2–17. 10.1093/nutrit/nuw043 27974596 PMC5155614

[27] Rutten M Achterbosch TJ de Boer IJM Cuaresma JC Geleijnse JM Havlík P The vision of the SUSFANS project. Agr Syst. (2019) 163:45–57. 10.1016/j.agsy.2016.10.014

[28] Van Dooren C. A review of the use of linear programming to optimize diets, nutritiously, economically and environmentally. Front Nutr. (2018) 5:48. 10.3389/fnut.2018.00048 29977894 PMC6021504

[29] Dantzig GB The diet problem. Interfaces. (1990) 20:43–47. 10.1287/inte.20.4.43

[30] Ferguson EL Darmon N Briend A Premachandra IM. Food-based dietary guidelines can be developed and tested using linear programming analysis. J Nutr. (2004) 134:951–7. 10.1093/jn/134.4.951 15051853

[31] Perignon M Masset G Ferrari G Barré T Vieux F Maillot M. How low can dietary greenhouse gas emissions be reduced without impairing nutritional adequacy, affordability and acceptability of the diet? A modelling study to guide sustainable food choices. Public Health Nutr. (2016) 19:2662–74. 10.1017/S1368980016000653 27049598 PMC10448381

[32] Horgan GW Perrin A Whybrow S Macdiarmid JI. Achieving dietary recommendations and reducing greenhouse gas emissions: modelling diets to minimise the change from current intakes. Int J Behav Nutr Phys Act. (2016) 13:46. 10.1186/s12966-016-0370-1 27056829 PMC4823893

[33] Donini LM Dernini S Lairon D Serra-Majem L Amiot M-J. A consensus proposal for nutritional indicators to assess the sustainability of a healthy diet: the mediterranean diet as a case study. Front Nutr. (2016) 3:37. 10.3389/fnut.2016.00037 27622186 PMC5002406

[34] Macdiarmid JI Kyle J Horgan GW Loe J Fyfe C Johnstone A. Sustainable diets for thefuture: can we contribute to reducing greenhouse gas emissions by eating a healthy diet. Am J Clin Nutr. (2012) 96:632–9. 10.3945/ajcn.112.038729 22854399

[35] Saxe H Larsen TM Mogensen L The global warming potential of two healthy nordic diets compared with the average danish diet. Clim Change. (2012) 116:249–62. 10.1007/s10584-012-0495-4

[36] Notarnica E Tassielli G Renzulli PA Castellani V Sala S Environmental impacts of food consumption in Europe. J Clean Prod. (2017) 140:753–65. 10.1016/j.jclepro.2016.06.080

[37] Vieux F Perignon M Gazan R Darmon N. Dietary changes needed to improve diet sustainability: are they similar across Europe? Eur J Clin Nutr. (2018) 72:951–60. 10.1038/s41430-017-0080-z 29402959 PMC6035144

[38] Bertoluci G Masset G Gomy C Mottet G Darmon N. How to build a standardized country-specific environmental food database for nutritional epidemiology studies. PLoS ONE. (2016) 11:e0150617. 10.1371/journal.pone.0150617 27054565 PMC4824438

[39] Donati M Menozzi D Zighetti C Rosi A Zinetti A Scazzina F. Towards a sustainable diet combining economic, environmental and nutritional objectives. Appetite. (2016) 106:48–57. 10.1016/j.appet.2016.02.151 26921487

[40] Sette S Le Donne C Piccinelli R Arcella D Turrini A Leclercq C. The third Italian national food consumption survey, INRAN-SCAI 2005–06 – Part 1: nutrient intakes in Italy. Nutr Metab Cardiovasc Dis. (2011) 21:922–32. 10.1016/j.numecd.2010.03.001 20674305

[41] Myhre G Shindell D Bréon FM Collins W Fuglestvedt J Huang J Anthropogenic and natural radiative forcing. In: Stocker TF Qin D Plattner G-K Tignor M Allen SK Boschung J, editors. Climate Change: The Physical Science Basis. Contribution of Working Group I to the Fifth Assessment Report of the Intergovernmental on Climate Change Cambridge University Press. Cambridge; New York, NY: Cambridge University Press (2013). p. 661–740.

[42] Hartikaiinen H Pulkkinen H Summary of the chosen methodologies and practices to produce GHGE-estimates for an average European diet. In: Natural Resources and Bioeconomy Studies 58/2016. Helsinki: Natural Resources Institute Finland (2016). p. 5–38.

[43] World Health Organization Guideline: Sugars Intake for Adults and Children. Geneva: WHO (2015). 25905159

[44] World Health Organization Diet, Nutrition and the Prevention of Chronic Diseases. Joint WHO/FAO Expert Consultation. WHO Technical Report Series no. 916. Geneva: WHO (2003). 12768890

[45] World Health Organization Global Status Report on Alcohol And Health. Geneva: WHO (2018).

[46] Anderson JJ Darwis NDM Mackay DF Celis-Morales CA Lyall DM Sattar N. Red and processed meat consumption and breast cancer: UK biobank cohort study and meta-analysis. Eur J Cancer. (2018) 90:73–82. 10.1016/j.ejca.2017.11.022 29274927

[47] Ferrari M Mistura L Patterson E Sjöström M Díaz LE Stehle P. Evaluation of iron status in European adolescents through biochemical iron indicators: the HELENA study. Eur J Clin Nutr. (2011) 65:340–9. 10.1038/ejcn.2010.279 21245877 PMC3049292

[48] Sette S Le Donne C Piccinelli R Mistura L Ferrari M Leclercq C. The third national food consumption survey, INRAN-SCAI 2005-06: major dietary sources of nutrients in Italy. Int J Food Sci Nutr. (2013) 64:1014–21. 10.3109/09637486.2013.816937 23865755

[49] Collings R Harvey LJ Hooper L Hurst R Brown TJ Ansett J. The absorption of iron from whole diets: a systematic review. Am J Clin Nutr. (2013) 98:65–81. 10.3945/ajcn.112.050609 23719560

[50] International Agency for Research on Cancer Methyleugenol. Vol. 101. Working Group on the Evaluation of Carcinogenic Risks to Humans. In: IARC Monographs on the Evaluation of Carcinogenic Risks to Humans. Lyon (2011).

[51] Garnett T Cooking up a storm: food, greenhouse gas emissions and our changing climate. In: Food Climate Research Network. Centre for Environmental Strategy; University of Surrey. (2008). p. 155.

[52] Saba A Turrini A Mistura G Cialfa E Vichi M Indagine nazionale sui consumi alimentari delle famiglie 1980-84: alcuni principali risultati. Riv Soc Ita Sc Alim. (1991) 19:53–65.

[53] Turrini A Saba A Perrone D Cialfa E D'Amicis A. Food Consumption patterns in Italy: the INN-CA Study 1994-96. Eur J Clin Nutr. (2001) 55:571–88. 10.1038/sj.ejcn.1601185 11464231

[54] SUSFANS Metrics, Models and Foresight for Sustainable Food and Nutritition Security. Available online at: https://www.susfans.eu/susfans-visualizer (accessed April 17, 2020).

[55] European Commission – Bioeconomy Food 2030. Available online at: https://ec.europa.eu/research/bioeconomy/index.cfm?pg=policy&lib=food2030 (accessed April 17, 2020).

[56] Smetana SM Bornkessel S Heinz V. A path from sustainable nutrition to nutritional sustainability of complex food systems. Front Nutr. (2019) 6:39. 10.3389/fnut.2019.00039 31032257 PMC6473629

[57] EC EU Agricultural Outlook for Markets and Income, 2018–2030. Brussels: European Commission; DG Agriculture and Rural Development.

[58] European Parliament(requested by DG-AGRI) Megatrends in the Agri-Food Sector: Global Overview and Possible Policy Response From an EU Perspective. Available online at: http://www.europarl.europa.eu/RegData/etudes/STUD/2019/629205/IPOL_STU(2019)629205_EN.pdf (accessed April 17, 2020).

[59] Masset G Soler LG Vieux F Darmon N. Identifying sustainable foods: the relationship between environmental impact, nutritional quality, and prices of foods representative of the French diet. J Acad Nutr Diet. (2014) 114:862–9. 10.1016/j.jand.2014.02.002 24703928

[60] Darmon N Drewnowski A. Contribution of food prices and diet cost to socioeconomic disparities in diet quality and health: a systematic review and analysis. Nutr Rev. (2015) 73:643–60. 10.1093/nutrit/nuv027 26307238 PMC4586446

[61] European Commission Environment Sustainable Food. (2016). Available online at: https://ec.europa.eu/environment/archives/eussd/food.htm (accessed April 17, 2020).

[62] Tirado-von der Pahlen C Sustainable diets for healthy people and a healthy planet. United nations system standing committee on nutrition. In: Discussion Paper (Los Angeles, CA). (2017). p. 36.

[63] Burlingame B Dernini S (editors). Sustainable Diets Linking Nutrition and Food System. CAB International: Wallingford; Boston, MA (2019). p. 280.

