# Supplementary material for: Could Dietary Goals and Climate Change Mitigation Be Achieved Through Optimized Diet? The Experience of Modeling the National Food Consumption Data in Italy
Source: Front Nutr. 2020 May 4;7:48. doi: 10.3389/fnut.2020.00048 (PMC7212824; doi:10.3389/fnut.2020.00048)
Supplement: Supplementary file 1 [file Data_Sheet_1.pdf]

Appendix 1 - Food product categories and subcategories, indicator products, GHGE-mean values and data points							
	**Categories**		**Subcategories**	**Items consumed**	**Mean of data points (kg CO_2e/kg of product)**	**Indicator product**	**Number of data points**
1	Cereals, cereal products and substitutes	1	Bread and flour	*All types of bread prepared with any type of flour (wheat, whole wheat, soya, maize, etc.), speciality breads, sponge bread, excl. breadcrumbs and toasted bread. All types of flour (wheat, corn, oat, couscous, millet, barley, rice, etc.) used in recipes (polenta, pizza, f, cakes), breadcrumbs. Plain white pizza, plain tomato pizza, excl. other pizzas which were codified through recipes*	1.00	Bread	2
					0.71	Wheat flour[1]	3
					0.45	Corn	1
					0.57	Barley	1
					0.57	Oat	1
					0.47	Millet	1
		2	Pizza	Pizza	1.30	Pizza	1
		3	Breakfast cereals	Breakfast cereals	1.85	Breakfast cereals	2
		4	Pasta with eggs, filled, etc.	*Fresh pasta with eggs, tortellini (fresh pasta with meat filling)*	1.10	Pasta with egg	1
		5	Pasta, pasta substitute and flour	*All types of pasta, incl. rice noodles, puff pastry dough.*	1.07	Pasta	5
		6	Rice	*All types of rice, white and brown, excl. rice in commercial rice pudding*	2.76	Rice	1
		7	Biscuits	*All types of sweet biscuits, incl. sugar-free biscuits*	1.40	Biscuits	6
					2.72	Croissant with cream[3]	1
					2.75	Croissant with jam[4]	1
		8	Cakes and sweet snacks		2.08	Snack cake[2]	1

		9	Savoury fine bakery products	*All types of crackers, breadsticks, rusk, popcorn, salty appetizers and savoury biscuits*	1.46	Crackers[5]	4
2	Pulses	10	Pulses, fresh or processed	*All types of pulses (fresh and processed): lentils, peas, chickpeas, all types of beans excl. green beans, soybeans*	0.78	Green bean	1
					0.50	Dried legumes	1
3	Vegetables	11	Leafy, fruiting and other vegetables, fresh	*Chicory, lettuce, spinach, etc. Fresh tomatoes used in salad and other recipes (e.g. tomato sauce). Aubergine, pepper, cucumber, pumpkin, courgette, spices and herbs, etc.*	0.30	Chicory	1
					0.45	Lettuce[6]	2
					0.10	Spinach[7]	1
					0.14	Carrots[8]	3
					1.35	Egg plant	1
					0.77	Tomatoes	2
					0.53	Cucumber	2
					0.70	Asparagus[9]	2
					1.10	Pumpkin	1
					1.00	Courgette	2
					0.24	Artichoke	1
					0.10	Mushroom[10]	1
					0.60	Broccolo[11]	2
		12	Roots and onions, fresh	*Onion, garlic, turnip-rooted celery, beetroot, daikon*	0.11	Onion[12]	4
		13	Vegetables, processed	*Vegetable preserves (in vinegar, oil or brine), canned tomatoes, vegetable soup dehydrated, industrial mixed salad with mayonnaise*	1.23	Canned tomatoes	2
		14	Spices and herbs	*Spices and herbs*	0.91	Pepper[13]	1
4	Potatoes & tapioca	15	Potatoes & potatoes-based	*Potatoes raw, sweet potatoes, tapioca*	0.13	Potatoes	4

			dishes & tapioca, excl. crisps				
		16	Potatoes crisp	*potato chips, potato chips with cheese, potato croquettes, potato flakes dehydrate*			
5	Fruit	17	Citrus fruits and other fruits	*Orange, lemon, grapefruit, tangerine, tangelo and all other types of fruit: apple, pear, peach, apricot, grape, fig, melon, watermelon, cherries, berries.*	0.25	Orange[14]	2
					0.24	Lemon[15]	2
					0.36	Mandarin	1
					0.14	Apple[16]	4
					0.31	Pear	2
					0.24	Peach[17]	1
					0.24	Apricot	1
					0.90	Grapefruit	1
					0.30	Fig[18]	1
					0.60	Strawberry	1
					0.40	Raspberry	1
					0.44	Blueberry	1
					0.49	Kiwi	2
					1.50	Melon	1
					0.24	Cherries[19]	1
		18	Exotic Fruits	*Banana, cherimoya, avocado, papaya, mango, lychee, pineapple*	1.35	Banana	2
					0.88	Avocado[20]	1
					1.84	Pineapple	2
		19	Nuts, seeds, dried fruit, olives and their products	*Nuts roasted, dried, in powder or in puree (almond, chestnut, walnut, coconut, pine nut, peanut, pistachio), seeds (pumpkin seed) , olives*	1.79	Walnut, hazelnut	2
					0.52	Olive	2
		20	Canned fruit	*Canned fruit*	1.23	Canned tomatoes	2

6	Meat, meat products and substitutes	21	Beef & veal, not preserved, excl. offal	*Beef, veal, industrial meat sauce*	15.45	Beef	2
		22	Pork, not preserved, excl. offal	*Pork meat, pork meat roasted (porchetta), foot pork raw, excl. offal*	3.65	Pork	1
		23	Poultry and game, not preserved, excl. offal	*Pheasant, chicken, roast chicken, goose, quail, turkey, ostrich, incl. offal*	1.88	Poultry	1
		24	Processed meat	*Ham, salami, sausages and other preserved meats, excl. offal*	15.03	Processed milk	1
		25	Other meats, not preserved, excl. offal	*Lamb, goat, mutton, kid, horse, donkey, lean deer, boar, rabbit, frog, land snails*	13.85	Lamb[21]	3
					1.40	Horse	1
					2.70	Rabbit	1
		26	Meat substitute	*Meat imitates*	0,46	Soya	1
		27	Offal, blood and their product	-	-	-	-
7	Fish and seafood	28	Crustaceans, shellfish, mussels	*All types of fish, molluscs, crustaceans, raw (fresh or frozen or preserved) and fish fingers*	3.00	Shrimps[22]	1
					6.70	Mussels[23]	1
		29	Fish, fresh	*All other types of fresh fish (fresh or frozen or preserved)*	2.27	Small pelagics[24]	1
					3.10	Cod	2
					3.03	Salmon[25]	3
					2.27	Ground fish[26]	1
		30	Fish, preserved		3.03	Salmon[25]	3
8	Milk, milk products and their substitutes	31	Milk, milk-based beverages	*All types of milk (liquid, condensed and powder form), milk substitute*	1.31	Cow milk	5
					1.15	Goat milk	2
					8.20	Powdered milk	1
					1.10	Soya drink[27]	2

		32	Yoghurt and fermented milk	*Yoghurt, drinkable yoghurt*	1.50	Yoghurt	2
		33	Milk based dessert and substitute	*Yoghurt-based dessert*	1.50	Yoghurt	2
		34	Cheese and substitutes	*All types of cheese (e.g. mozzarella, parmesan, edam, feta, fontina), flavoured sweet cheese for children*	4.60	Seasoned cheese[28]	1
					25.20	Sheep cheese[29]	1
					1.68	Soft cheese[30]	1
					10.14	Mozzarella	2
					14.00	Swiss cheese	1
					5.87	Hard cheese[31]	2
					2.52	Ricotta	2
9	Oils & Fats	35	Olive oil	*Extra-virgin olive oil, olive oil, incl. enriched type*	5.12	Olive oil	2
		36	Other vegetable oil	*All type of vegetable oils, excl. olive oil*	1.73	Vegetable corn oil	1
					1.93	Vegetable sunflower oil	1
					0.95	Vegetable peanut oil	1
		37	Butter, creams and other fats	*Butter, cream, heavy cream, margarine all types, mayonnaise, lard*	3.36	Butter	1
					6.00	Cream	1
		38	Other fats	*soybean oil and butter*	2.52	Vegetable soy oil	1
10	Sweet products and substitutes	39	Ice cream, ice lolly and substitutes	*All types of ice creams (with all possible flavours and ingredients), ice lolly*	4.14	Ice cream	1
		40	Chocolate and substitutes	*All types of chocolate (milk, white, cocoa 70–90%, with hazelnuts, etc.), spreadable chocolate cream with or without hazelnuts, chocolate bars filled with toffee*	4.2	Chocolate	1

		41	Sugar, fructose, honey and other nutritious sweetener	*Honey, sugar, fructose, maple syrup, royal jell*	1.63	Sugar	2
					2,09	Honey	1
		42	Candies, jam and other sweet products	*All types of candies (e.g. toffee, fudge, fondant, different flavours), chewing gum, jam, marmalade, nougat with almonds, glazed chestnuts (marrons glacé),*	1.63	Sugar	2
		43	Cacao and cacao-based powder	*Cacao and cacao-based powder*	4.2	Chocolate	1
		44	Artificial sweeteners	*Artificial sweeteners*	1.63	Sugar	2
11	Meal substitute	45	Meal substitute	Meal substitute drink	4.2	Chocolate	1
12	Eggs	46	Eggs	*All types of eggs (chicken, duck, ostrich, etc.) excl. fish egg*	2.7	Egg	1
13	Non-alcoholic beverages	47	Tap water (as such, in beverages or recipes)	*Tap water*	< 0.01	Drinking water	2
		48	Mineral water	*All types of commercial bottled water*	0.21	Bottled water	2
		49	Herbal tea, tea, coffee and substitutes (decaffeinated)	*All types of coffee (e.g. brewed, decaffeinated, 'espresso', powdered), all types of tea (powdered, brewed, decaffeinated) with or without sugar, herbal tea (e.g. Camomile), pearled barley coffee, iced tea*	1.43	Coffee	1
					3.10	Tea	4
		50	Fruit & vegetable juices (without artificial sweetener)	*All types of fruit and vegetable juices (e.g. nectar, carrot juice, orange juice), with or without the addition of water, sugar and intense sweeteners*	0.67	Orange juice	1

		51	Other soft drinks	*All types of carbonate beverages (e.g. cola, soda, ginger ale, orange, tonic water) with sugar or sugar free, energy drinks, sport drinks, syrups to be diluted (incl. almond milk)*	0.25	Coke	1
14	Miscellaneous	52	Miscellaneous	*Miscellaneous*	0.25	Coke	1
15	Alcoholic beverages	53	Regular wine and substitute	*All type of wine*	1.39	Wine	3
		54	Sweet wine, spumante, wine-based aperitives & liquor	*Sweet wine, spumante, wine-based aperitives & liquor*	1.50	Spirits	1
		55	Beer, cider and substitute	*Beer, cider and substitute*	1.11	Beer	1

[1]semolina, spelt, bran, cous-cous, rye flour; [2] included sponge cake, puff pastry; [3] included cake, pastry; [4]included cake with jam; [5] included melba toast, michetti, tarallucci, grissini; [6] included salad, cardoon, seaweed, endue, radicchio, rocket, nettle; [7] included agretti, chard;[8] included fennel, celeriac, ginseng, ref been; [9] included witloof; [10] included truffle; [11] included cauliflower, turnip tops, brussels sprout, head cabbage; [12] included garlic, leek; [13] included spices and herbs, parsley, basil, rosemary, caper; [14] included candied fruit;[15] included grapefruit, tangelo, tangerine; [16] included pomegranate, quince, loquat; [17] included persimmon, plum; [18] included dates; [19] included black cherry; [20] included mango, lychee, cherimoya; [21] included goat; [22] included lobster, crab meat, spiny spider crab; [23] included squid, oyster, octopus, cuttlefish, clam, scallop; [24] included anchovy, herring, flathead mullet, snapper, mackerel, mullus surmuletus, trout, gilthead sea, swordfish; [25] included caviar; [26] included stock fish, big scale sand melt, tuna, smooth dagfish, rays, turbot, sole, sea bass, grouper, perch, salema, tub gurnard; [27] included rice milk, tofu; [28] included “bel paese”, caciocavallo, caciotta, provolone, scamorza, goat cheese, camembert, brie, “certosino”, vezzena, provola; [29] included pecorino; [30] included stracchino, processed cheese, gorgonzola, latteria, mascarpone, “Philadelphia”, fresh cottage cheese, singles pasteurized cheese, robiola, feta, burrata; [31] included parmesan.
