# Supplementary material for: Could Dietary Goals and Climate Change Mitigation Be Achieved Through Optimized Diet? The Experience of Modeling the National Food Consumption Data in Italy
Source: Front Nutr. 2020 May 4;7:48. doi: 10.3389/fnut.2020.00048 (PMC7212824; doi:10.3389/fnut.2020.00048)
Supplement: Supplementary file 2 [file Data_Sheet_2.pdf]

| Appendix 2 - Literature references used for GHGE data points of indicator products | | | | | | |
|---|---|---|---|---|---|---|
| | | | | | | |
| *n* | **Indicator product** | **Authors** | **Year of Publication** | **Journal** | **Type of publication** | **Analysis location** |
| **1** | **Bread** | Espinoza-Orias N, Stichnothe H, Azapagic A | 2011 | The International Journal of Life Cycle Assessement | Scientific paper | United Kingdom |
| | | Korsaeth A, Jacobsen AZ, Roer AG, Henriksen TM, Sonesson U, Bonesmo H, Skjelvåg AO, Strømman A | 2012 | Acta Agriculturae Scandinavica, Section A — Animal Science | Scientific paper | Norway |
| **2** | **Wheat flour** | Fantin V, Rondini I, Righi S, Pasteris A, Coatti F, Passerini F, Masoni P | 2015 | Proceedings of the International conference on Life Cycle Assessment as reference methodology for assessing supply chains and supporting global sustainability challenge - LCA for “Feeding the planet and energy for life” EXPO 2015 | Conference paper | Italy |
| | | Ruini L, Ferrari E, Meriggi P, Marino M, Sessa S, Principato L | 2013 | Agriregionieuropa | Report | Italy |
| | | Castaldi S, Fidaleo M, Moresi M, Valentini R | 2009 | University of Tuscia, Italy | Report | Italy |
| **3** | **Mais** | Fantin V, Rondini I, Righi S, Pasteris A, Coatti F, Passerini F, Masoni P | 2015 | Proceedings of the International conference on Life Cycle Assessment as reference methodology for assessing supply chains and supporting global sustainability challenge - LCA for “Feeding the planet and energy for life” EXPO 2015 | Conference paper | Italy |
| **4** | **Barley** | Rajaniemi M,Mikkola H, Ahokas J | 2011 | Agronomy Research | Scientific paper | Finland |
| **5** | **Oat** | Rajaniemi M,Mikkola H, Ahokas J | 2011 | Agronomy Research | Scientific paper | Finland |
| **6** | **Millet** | Audsley E, Brander M, Chatterton J, Murphy-Bokern D, Webster C, Williams A | 2009 | WWF-UK | Report | United Kingdom |

| | | | | | | |
|---|---|---|---|---|---|---|
| **7** | **Breakfast cereal** | Jeswani HK, Burkinshaw R, Azapagic A | 2015 | Sustainable production and consumption | Scientific paper | United Kingdom |
| | | Ma BL, Liang, BC, Biswas DK. | 2012 | Nutrient Cycling in Agroecosystems | Scientific paper | Canada |
| **8** | **Pizza** | National Footprint Account | 2008 | Global Footprint Network | Report | Italy |
| **9** | **Pasta with egg** | Ministry of the Environment and Protection of Land and Sea | 2013 | National programme for carbon footprint assessment | Report | Italy |
| **10** | **Pasta** | Ruini L, Ferrari E, Meriggi P, Marino M, Sessa F, Principato L | 2013 | Agriregionieuropa | Scientific paper | Italy |
| | | Ministry of the Environment and Protection of Land and Sea | 2015 | National programme for carbon footprint assessment | Report | Italy |
| | | Ministry of the Environment and Protection of Land and Sea | 2011 | National programme for carbon footprint assessment | Report | Italy |
| | | Ministry of the Environment and Protection of Land and Sea | 2014 | National programme for carbon footprint assessment | Report | Italy |
| | | Voiello, Registration number S-P 00491 | 2014 | Environmental declaration | Technical sheet | Italy |
| **11** | **Rice** | Blengini GA, Busto M | 2009 | Journal of Evironmental Management | Scientific paper | Italy |
| **12** | **Biscuits** | Barilla, Registration number S-P 00421 | 2005 | Environmental declaration | Technical sheet | Italy |
| | | Barilla, Registration number S-P-00324 | 2012 | Environmental declaration | Technical sheet | Italy |
| | | Barilla, Registration number S-P 00485 | 2013 | Environmental declaration | Technical sheet | Italy |
| | | Barilla, Registration number S-P-00230 | 2012 | Environmental declaration | Technical sheet | Italy |
| | | Barilla, Registration number S-P 00416 | 2013 | Environmental declaration | Technical sheet | Italy |
| | | Castaldi S, Fidaleo M, Moresi M, Valentini R | 2009 | University of Tuscia. Italy | Report | Italy |
| **13** | **Snack cake** | National Footprint Account | 2008 | Global Footprint Network | Report | Italy |
| **14** | **Croissant with cream** | Barilla, Registration number S-P-00319 | 2012 | Environmental declaration | Technical sheet | Italy |
| **15** | **Croissant with jam** | Barilla, Registration number S-P-00330 | 2012 | Environmental declaration | Technical sheet | Italy |
| **16** | **Crackers** | Barilla, Registration number S-P-00229 | 2013 | Environmental declaration | Technical sheet | Italy |
| | | Barilla, Registration number S-P-00225 | 2013 | Environmental declaration | Technical sheet | Italy |
| | | Barilla, Registration number S-P-00331 | 2012 | Environmental declaration | Technical sheet | Italy |
| | | Barilla, Registration number S-P-00226 | 2013 | Environmental declaration | Technical sheet | Italy |

| | | | | | | |
|---|---|---|---|---|---|---|
| **17** | **Green bean** | National Footprint Account | 2008 | Global Footprint Network | Report | Italy |
| **18** | **Dried legumes** | Fuentes C, Carlsson-Kanyama A, Biel A, Bergström K, Carlsson-Kanyama A, Fuentes C, Grankvist G, Lagerberg G, Fogelberg C, Shanahan H, Solér C | 2006 | Environ Management | Scientific paper | United Kingdom |
| **19** | **Chicory** | Tamburini E, Pedrini P, Marchetti G, Fano EA, Castaldelli G | 2015 | Sustainability | Scientific paper | Italy |
| **20** | **Lettuce** | Bartzas G, Zaharaki D, Komnitsas K | 2015 | Information processing in agriculture | Scientific paper | Italy and Spain |
| | | Castaldi S, Fidaleo M, Moresi M, Valentini R. | 2009 | University of Tuscia. Italy | Report | Italy |
| **21** | **Carrots** | Castaldi S, Fidaleo M, Moresi M, Valentini R | 2009 | University of Tuscia. Italy | Report | Italy |
| | | Raghu KC | 2014 | Lappeenranta University of Technology, Master thesis | Master thesis | Finland |
| | | Karlsson H | 2012 | Norwegian University of Life Science | Master thesis | Norway |
| **22** | **Egg plant** | Audsley E, Brander M, Chatterton J, Murphy-Bokern D, Webster C, Williams A | 2009 | WWF-UK | Report | United Kingdom |
| **23** | **Spinach** | Kramer KJ, Moll HC, Nonhebel S | 1999 | Agriculture, ecosystem and Environment | Scientific paper | The Netherlands |
| **24** | **Tomatoes** | Cellura M , Ardente F, Longo S | 2012 | Journal of Environmental Management | Scientific paper | Italy |
| | | Theurl MC, Haberl H, Erb KH, Lindenthal T | 2014 | Agronomy for Sustainable Development | Scientific paper | Italy |
| **25** | **Canned tomatoes** | Castaldi S, Fidaleo M, Moresi M, Valentini R | 2009 | University of Tuscia. Italy | Report | Italy |
| | | Manfredi M, Vignali G | 2014 | Journal of Cleaner Production | Scientific paper | Italy |
| **26** | **Pepper** | Cellura M , Ardente F, Longo S | 2012 | Journal of Environmental Management | Scientific paper | Italy |
| **27** | **Cucumber** | Audsley E, Brander M, Chatterton J, Murphy-Bokern D, Webster C, Williams A | 2009 | World Wildlife Fund (WWF) and Food Climate research network WWF-UK | Report | United Kingdom |
| | | Raab M, Brunklaus B | 2012 | Chalmers University of Technology | Scientific paper | Spain |
| **28** | **Asparagus** | Soode E, Lampert P, Weber-Blaschke G, Richter K | 2014 | Journal of Cleaner Production | Scientific paper | Germany |
| | | Blanke M, Schaefer F | 2012 | University of Bonn | Lecture | Germany |
| **29** | **Pumpkin** | Blanke M, Schaefer F | 2012 | University of Bonn | Lecture | Germany |

| | | | | | | |
|---|---|---|---|---|---|---|
| **30** | **Courgette** | Castaldi S, Fidaleo M, Moresi M, Valentini R | 2009 | University of Tuscia. Italy | Report | Italy |
| | | Cellura M , Ardente F, Longo S | 2012 | Journal of Environmental Management | Scientific paper | Italy |
| **31** | **Broccolo** | Edwards J, Plassmann EJ, Hounsome Y, Mila i Canals J | 2008 | Environmental Science & Policy. | Scientific paper | United Kingdom |
| | | DEFRA | 2014 | T H Clements's Carbon Footprinting Challenge | Report | United Kingdom |
| **32** | **Artichoke** | Lo Giudice A, Mbohwa C, Clasadonte MT, Ingrao C | 2014 | International Journal of Environmental Research | Scientific paper | Italy |
| **33** | **Mushroom** | Audsley E, Brander M, Chatterton J, Murphy-Bokern D, Webster C, Williams A | 2009 | World Wildlife Fund (WWF) and Food Climate research network WWF-UK | Report | United Kingdom |
| **34** | **Onion** | Fogelberg C, Carlsson-Kanyama A | 2006 | Swedish Defence Agency | Report | Sweden |
| | | O'Halloran, Fisher and Rab | 2008 | Horticulture Australia Limited (HAL) | Conference paper | Australia |
| | | Fogelberg C, CarlssonKanyama A | 2006 | Swedish Defence Agency | Report | Denmark Sweden |
| | | Saunders C, Barber A, Taylor G | 2006 | Lincoln University | Report | United Kingdom |
| **35** | **Potatoes** | Carlsson-Kanyama A | 1998 | Food Policy | Scientific paper | Sweden |
| | | Ecoinvent Centre | 2013 | Swiss Centre for Life Cycle Inventories | Report | Switzerland |
| | | Kramer KJ, Moll HC, Nonhebel S | 1999 | Agriculture Ecosystem & Environment | Scientific paper | The Netherlands |
| | | Röös E, Sundberg C, Hansson PA | 2010 | International Journal of Life Cycle Assessment | Scientific paper | United Kingdom |
| **36** | **Orange** | Pergola M, D'Amico M, Celano G, Palese AM, Scuderi A, Di Vita G, Pappalardo G, Inglese P | 2013 | Journal of Environmental Management | Scientific paper | Italy |
| | | Castaldi S, Fidaleo M, Moresi M, Valentini R. | 2009 | University of Tuscia. Italy | Report | Italy |
| **37** | **Lemon** | Pergola M, D'Amico M, Celano G, Palese AM, Scuderi A, Di Vita G, Pappalardo G, Inglese P | 2013 | Journal of Environmental Management | Scientific paper | Italy |
| | | Castaldi S, Fidaleo M, Moresi M, Valentini R | 2009 | University of Tuscia. Italy | Report | Italy |

| | | | | | | |
|---|---|---|---|---|---|---|
| **38** | **Mandarin** | Castaldi S, Fidaleo M, Moresi M, Valentini R | 2009 | University of Tuscia. Italy | Report | Italy |
| **39** | **Apple** | Sessa F, Marino M, Montanaro G, Dal Piaz A, Zanotelli D,<br>Mazzetto F, Tagliavini M | 2014 | Proceedings of the 9th International Conference on Life Cycle Assessment in the Agri-Food Sector | Conference paper | Italy |
| | | Castaldi S, Fidaleo M, Moresi M, Valentini R | 2009 | University of Tuscia. Italy | Report | Italy |
| | | Tamburini E , Pedrini P , Marchetti MG , Fano EA and Castaldelli G. | 2015 | Sustainability | Scientific paper | Italy |
| | | Cerutti AK, Bruun S, Donno D, Beccaro GL, Bounous G | 2013 | Journal of Cleaner Production | Scientific paper | Italy |
| **40** | **Pear** | Castaldi S, Fidaleo M, Moresi M, Valentini R | 2009 | University of Tuscia. Italy | Report | Italy |
| | | Tamburini E , Pedrini P , Marchetti MG , Fano EA, Castaldelli G | 2015 | Sustainability | Scientific paper | Italy |
| **41** | **Peach** | Castaldi S, Fidaleo M, Moresi M, Valentini R | 2009 | University of Tuscia. Italy | Report | Italy |
| **42** | **Apricot** | Castaldi S, Fidaleo M, Moresi M, Valentini R | 2009 | University of Tuscia. Italy | Report | Italy |
| **43** | **Grapefruit** | Castaldi S, Fidaleo M, Moresi M, Valentini R | 2009 | University of Tuscia. Italy | Report | Italy |
| **44** | **Fig** | National Footprint Account | 2008 | Global Footprint Network | Report | Italy |
| **45** | **Strawberry** | REWE Group | 2009 | Product Carbon Footprint | Report | Spain |
| | | Williams A, Pell E, Webb J, Moorhouse E, Audsley E | 2008 | Proceedings of the 6th International Conference on LCA in the Agri-Food Sector | Conference paper | Spain |
| | | Williams A, Pell E, Webb J, Moorhouse E, Audsley E | 2008 | Proceedings of the 6th International Conference on LCA in the Agri-Food Sector | Conference paper | United Kingdom |
| | | Lillywhite R | 2008 | University of Warwick. United Kingdom | Report | United Kingdom |
| **46** | **Raspberry** | Girgenti V, Peano C, Baudino C, Tecco N | 2014 | Science of the Total Environment | Scientific paper | Italy |
| **47** | **Bluesberry** | Girgenti V, Peano C, Baudino C, Tecco N | 2014 | Science of the Total Environment | Scientific paper | Italy |
| **48** | **Cherries** | Castaldi S, Fidaleo M, Moresi M, Valentini R | 2009 | University of Tuscia. Italy | Report | Italy |
| **49** | **Melon** | Castaldi S, Fidaleo M, Moresi M, Valentini R | 2009 | University of Tuscia. Italy | Report | Italy |

| | | | | | | |
|---|---|---|---|---|---|---|
| **50** | **Kiwi** | Audsley E, Brander M, Chatterton J, Murphy-Bokern D, Webster C, Williams A | 2009 | World Wildlife Fund (WWF) and Food Climate research network WWF-UK | Report | United Kingdom |
| | | Robertson K, Garnham M, Symes W | 2014 | Int J Life Cycle Asses | Scientific paper | New Zeland |
| **51** | **Banana** | Audsley E, Brander M, Chatterton J, Murphy-Bokern D, Webster C, Williams A | 2009 | World Wildlife Fund (WWF) and Food Climate research network WWF-UK | Report | United Kingdom |
| | | Svanes E,  Aronsson Anna KS | 2013 | The International Journal of Life Cycle Assessment | Scientific paper | Costa Rica |
| **52** | **Avocado** | Audsley E, Brander M, Chatterton J, Murphy-Bokern D, Webster C, Williams A | 2009 | World Wildlife Fund (WWF) and Food Climate research network WWF-UK | Report | United Kingdom |
| **53** | **Pineapple** | Moss R | 2011 | West Africa Fair Fruit (WAFF) | Report | Ghana |
| | | Audsley E, Brander M, Chatterton J, Murphy-Bokern D, Webster C, Williams A | 2009 | World Wildlife Fund (WWF) and Food Climate research network WWF-UK | Report | United Kingdom |
| **54** | **Walnut, hazelnut** | Volpe R, Messineo S, Volpe M, Messineo A. | 2015 | Sustainability | Scientific paper | Italy |
| | | National Footprint Account | 2008 | Global Footprint Network | Report | Italy |
| **55** | **Olive** | Rinaldi S, Barbanera M, Lascaro E | 2014 | Science of the total Environment | Scientific paper | Italy |
| | | De Gennaro B, Notarnicola B, Roselli L | 2012 | Journal of Cleaner Production | Scientific paper | Italy |
| **56** | **Beef** | Coderoni S, Sonaglia L | 2014 | MIPAAF-INEA | National report | Italy |
| | | Castaldi S, Fidaleo M, Moresi M, Valentini R | 2009 | University of Tuscia. Italy | Report | Italy |
| **57** | **Poultry** | Coderoni S, Sonaglia L | 2014 | MiPAAF-INEA | National report | Italy |
| **58** | **Pork** | Coderoni S, Sonaglia L | 2014 | MiPAAF-INEA | National report | Italy |
| **59** | **Lamb** | Jones AK, Jones DC, Cross P | 2014 | Agricultural Systems | Scientific paper | United Kingdom |
| | | Jones AK, Jones DC, Cross P | 2014 | Agricutural Systems | Scientific paper | United Kingdom |
| | | Jones AK, Jones DC, Cross P | 2014 | Agricultural Systems | Scientific paper | United Kingdom |

| | | | | | | |
|---|---|---|---|---|---|---|
| **60** | **Horse** | Ferrari M, Sette S, Mistura L, Le Donne C, Piccinelli R, Buonocore P, Leclercq C | 2010 | Public Health Nutrition | Conference abstract | Italy |
| **61** | **Processed meat** | BCNF | 2013 | Double Pyramid | Report | Italy |
| **62** | **Rabbit** | Ferrari M, Sette S, Mistura L, Le Donne C, Piccinelli R, Buonocore P, Leclercq C | 2010 | Public Health Nutrition | Conference abstract | Italy |
| **63** | **Soia** | Gonzalez A, Frostell A, Carlsson-Kanyama | 2011 | Food Policy | Scientific paper | United Kingdom |
| **64** | **Shrimps** | LCA Food Database | 2008 | www.lcafood.dk | Database | Denmark |
| **65** | **Mussels** | LCA Food Database | 2008 | www.lcafood.dk | Database | Denmark |
| **66** | **Small pelagics** | Iribarren D, Vázquez-Rowe I, Hospido A, Moreira MT, Feijoo G. | 2011 | Sci Total Environ | Scientific paper | Italy |
| **67** | **Cod** | Smárason BÖ, Vidarsson JR, Þórðarson G, Magnúsdóttir L | 2014 | Matis | Scientific paper | United Kingdom from Iceland |
| | | LCA Food Database | 2008 | www.lcafood.dk | Database | Denmark |
| **68** | **Salmon** | Ziegler F, Winther U, Hognes ES, Emanuelsson A, Sund V, Ellingsen H | 2013 | Journal of Industrial Ecology | Scientific paper | Norway to Paris |
| | | LCA Food Database | 2008 | www.lcafood.dk | Database | Denmark |
| | | Pelletier, N, Tyedmers P, Sonelsson U, Scholz A, Ziegler F, Flysjo A, Kruse S, Cancino B, Silverman H | 2009 | Environmental Science & Technology | Scientific report | Chile, Canada, United Kingdom |
| **69** | **Ground fish** | Castaldi S, Fidaleo M, Moresi M, Valentini R | 2009 | University of Tuscia. Italy | Report | Italy |
| **70** | **Cow milk** | Fantin V, Buttol P, Pergreffi R, Masoni P | 2012 | Journal of Cleaner Production | Scientific paper | Italy |
| | | Penati CA, Tamburini A, Bava L, Zucali M, Sandrucci A | 2013 | Journal of Animal Science | Scientific paper | Italy |
| | | Bava L, Zucali M, Tamburini A, Guerci M, Sandrucci A | 2014 | Quaderni della Ricerca n. 163, Regione Lombardia, Italy | Scientific paper | Italy |
| | | Guerci M, Bava L, Zucali M, Tamburini A, Sandrucci A | 2014 | Journal of Cleaner Production | Scientific paper | Italy |

| | | | | | | |
|---|---|---|---|---|---|---|
| | | Guerci M, Bava L, Zucali M, Sandrucci A, Penati C, Tamburini A | 2013 | Journal of Dairy Research | Scientific paper | Italy |
| **71** | **Goat milk** | Kanyarushoki C, Fuchs F, van der Werf HMG | 2008 | Proceedings of the 6th International Conference on Life Cycle Assessment in the Agri-Food Sector | Conference paper | France |
| | | Robertson K, Symes W, Garnham M | 2015 | Journal of Dairy Science | Scientific paper | New Zealand |
| **72** | **Powered milk** | Flysjö A | 2012 | Aarhus University, Denmark | Scientific paper | Denmark |
| **73** | **Soya drink** | Blonk H, Kool A, Luske B, de Waart S | 2008 | Blonk Milieu Advies B.V. | Scientific paper | The Netherlands |
| | | Ogle SM, Del Grosso J, Adler PR, Parton WJ | 2009 | Lifecycle Carbon Footprint of Biofuels Workshop | Scientific paper | Finland |
| **74** | **Yogurt** | Vergé XP, Maxime D, Dyer JA, Desjardins RL,Arcand Y, Vanderzaag A | 2013 | Journal of Dairy Science | Scientific paper | Canada |
| | | González-García S, Castanheira EG, Dias AC, Arroj L | 2013 | International Journal of Life Cycle Assessment | Scientific paper | Portugal |
| **75** | **Seasoned cheese** | National Footprint Account | 2008 | Global Footprint Network | Report | Italy |
| **76** | **Sheep cheese** | Favilli A, Rizzi F, Iraldo F | 2003 | Parallel 1a: Life Cycle 2 Inventory, Databases and Tools | Conference paper | Italy |
| **77** | **Soft cheese** | Castaldi S, Fidaleo M, Moresi M, Valentini R | 2009 | University of Tuscia. Italy | Report | Italy |
| **78** | **Mozzarella** | Simonetto M, Mazzi A, Fedel A, Pieretto C, Scipioni A | 2015 | Proceedings of the International conference on Life Cycle Assessment as reference methodology for assessing supply chains and supporting global sustainability challenge - LCA for “Feeding the planet and energy for life” EXPO 2015 | Conference paper | Italy |
| | | Granarolo, Registration number S-P 00128 | 2014 | Environmental declaration | Technical sheet | Italy |
| **79** | **Swiss cheese** | Nenecek T, Schmid A, Alig M, Schnebli K, Vaihinger M | 2011 | Proceeding of SETAC Europe 17th LCA Case Studies Symposium | Conference paper | Switzerland |

| | | | | | | |
|---|---|---|---|---|---|---|
| **80** | **Hard cheese** | Caseificio Caramsche So c. Coop | 2015 | Environmental declaration | External Communication Report | Italy |
| | | Castaldi S, Fidaleo M, Moresi M, Valentini R | 2009 | University of Tuscia. Italy | Report | Italy |
| **81** | **Ricotta** | Simonetto M, Mazzi A, Fedel A, Pieretto C, Scipioni A | 2015 | Proceedings of the International conference on Life Cycle Assessment as reference methodology for assessing supply chains and supporting global sustainability challenge - LCA for “Feeding the planet and energy for life” EXPO 2015 | Conference paper | Italy |
| | | Castaldi S, Fidaleo M, Moresi M, Valentini R | 2009 | University of Tuscia. Italy | Report | Italy |
| **82** | **Olive oil** | Pattaraa C, Salomoneb R, Cichellia A | 2016 | Journal of Cleaner Production | Scientific paper | Italy |
| | | Castaldi S, Fidaleo M, Moresi M, Valentini R | 2009 | University of Tuscia. Italy | Report | Italy |
| **83** | **Vegetable mais oil** | Marchio Zucchi | 2016 | Environmental declaration | External Communication Report | Italy |
| **84** | **Vegetable soia oil** | Marchio Zucchi | 2016 | Environmental declaration | External Communication Report | Italy |
| **85** | **Vegetable sunflower oil** | Marchio Zucchi | 2016 | Environmental declaration | External Communication Report | Italy |
| **86** | **Vegetable peanut oil** | Marchio Zucchi | 2016 | Environmental declaration | External Communication Report | Italy |
| **87** | **Butter** | Castaldi S, Fidaleo M, Moresi M, Valentini R | 2009 | University of Tuscia. Italy | Report | Italy |
| **88** | **Cream** | Flysjö A | 2012 | Aarhus University. Denmark | PhD thesis | Denmark |
| **89** | **Ice cream** | Sammontana | 2017 | Environmental declaration | External Communication Report | Italy |

| | | | | | | |
|---|---|---|---|---|---|---|
| **90** | **Honey** | National Footprint Account | 2008 | Global Footprint Network | Report | Italy |
| **91** | **Chocolate** | National Footprint Account | 2008 | Global Footprint Network | Report | Italy |
| **92** | **Sugar** | Lapi G | 2013 | Università Bocconi, Milan, Italy | Conference paper | Italy |
| | | Castaldi S, Fidaleo M, Moresi M, Valentini R | 2009 | University of Tuscia. Italy | Report | Italy |
| **93** | **Eggs** | Taylor RC, Omed H, Edwards-Jones G. | 2014 | Poultry Science | Scientific paper | United Kingdom |
| | | Agri-food production in Alberta | 2014 | Alberta Agricultural and rural development | Report | Canada |
| **94** | **Drinking water** | Fantin V, Scalbi S, Masoni P | 2012 | VI Convegno della Rete Italiana LCA | Conference paper | Italy |
| | | Botto S | 2007 | Proceedings Nature | Scientific paper | Italy |
| **95** | **Bottled water** | Fantin V, Scalbi S, Masoni P | 2012 | VI Convegno della Rete Italiana LCA | Conference paper | Italy |
| | | Botto S | 2007 | Proceedings Nature | Scientific paper | Italy |
| **96** | **Coffee** | National Footprint Account | 2008 | Global Footprint Network | Report | Italy |
| **97** | **Tea** | Azapagic A | 2013 | Impact Assessment and Policy Action National Multi-Stakeholder Workshop | Conference paper | Kenya |
| | | AIT. Small and medium scale industries in Asia: energy and environmental tea sector | 2002 | Asian Institute of Technology | Report | Sri Lanka |
| | | Taulos JL, Sebtosi AB | 2016 | Renewable and Sustainable Energy Reviews | Scientific paper | Malawian |
| **98** | **Orange juice** | Beccalia M, Celluraa M, Iudicelloa M, Mistret M | 2010 | Journal of Environmental Management | Scientific paper | Italy |
| **99** | **Coca-cola** | Carbon trust | 2009 | Carbon trust | Technical sheet | United Kingdom |
| **100** | **Wine** | Bonamentea E, Scruccaa F, Rinaldia S, Mericoa MC, Asdrubalib F, Lamastra L | 2016 | Science of The Total Environment | Scientific paper | Italy |
| | | Bosco S, Di Benedetti C, Galli M, Remorini D, Massai R, Bonari E | 2016 | Italian Journal of Agronomy | Scientific paper | Italy |
| | | National Footprint Account | 2008 | Global Footprint Network | Report | Italy |
| **101** | **Spirits** | Consulting LLC | 2011 | Four Elements Seattle | Report | USA |

| | | | | | | |
|---|---|---|---|---|---|---|
| **102** | **Beer** | National Footprint Account | 2008 | Global Footprint Network | Report | Italy |
